# Dihydrotestosterone in Amyotrophic lateral sclerosis—The missing link?

**DOI:** 10.1002/brb3.1645

**Published:** 2020-10-13

**Authors:** Nishit Sawal, Jasbinder Kaur, Kamaljeet Kaur, Satinder Gombar

**Affiliations:** ^1^ Neurology Division, Department of Medicine Government Medical college and Hospital Chandigarh India; ^2^ Department of Biochemistry Government Medical college and Hospital Chandigarh India; ^3^ Department of Anaesthesia and Intensive care Government Medical college and Hospital Chandigarh India

**Keywords:** Amyotrophic Lateral Sclerosis, blood–brain barrier, Cerebrospinal fluid, dihydrotestosterone, Luteinizing hormone, sex hormone binding globulin

## Abstract

**Objective:**

Testosterone has been postulated to be involved in ALS causation.

**Materials and methods:**

CSF levels of free testosterone and dihydrotestosterone were measured in 13 ALS patients [7 males, 6 females] and 22 controls [12 males, 10 females].

**Results:**

CSF free testosterone levels did not show any significant differences but CSF dihydrotestosterone levels were significantly decreased in all male and female ALS patients.

**Conclusions:**

DHT is probably integral to survival of motor neurons. In patients predisposed to develop ALS, there is possibly a sort of “testosterone resistance” at level of blood–brain barrier [BBB] existing right from birth and is likely the result of dysfunctional transport protein involved in testosterone transfer across the BBB. In these patients, lesser amount of testosterone is able to breach the BBB and enter the central neural axis. Lesser amount of testosterone is available to 5 α reductase in the anterior pituitary to be converted to DHT and lesser amount of DHT is generated. There is inadequate negative feedback suppression of LH at the level of anterior pituitary by DHT. As a result of higher LH levels, testosterone levels rise in the peripheral testosterone fraction [the fraction outside the BBB] and this explains the various physical attributes of ALS patients like lower Ratio of the index and ring finger lengths (2D:4D ratio), increased incidence of early onset alopecia etc. This deficiency of DHT leads to motor neuron death causing ALS.

## Graphical abstract

CSF levels of dihydrotestosterone were measured in 13 ALS patients [7 males and 6 females] and in 22 controls [12 males and 10 females], and we found that CSF dihydrotestosterone levels were significantly decreased in all the male and female ALS patients dihydrotestosterone probably plays a key role in ALS pathogenesis.

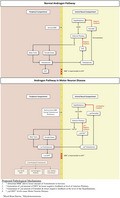

## 1 INTRODUCTION

Amyotrophic lateral sclerosis is a progressive neurodegenerative disorder characterized by affliction of both the upper and lower motor neurons.(Chio et al., 2009; Cozzolino, Ferri, & Teresa Carrì, 2008; Manjaly et al., 2010; Worms, 2001) It has been postulated that androgens/testosterone may play some role in causation of ALS (Weiner, 1980 ; Wicks, 2012) based on the following observations—Male‐to‐female ratio. There have been frequent reports of preponderance of male to female patients with ALS (Manjaly et al., 2010). Sparing of neurons of cranial nerves III, IV, and VI and Onuf's nucleus in ALS that lack androgen receptors and are not involved even in advanced MND (Weiner, 1980). A type of MND—X‐linked spinobulbar muscular atrophy (Kennedy's disease), results from a trinucleotide repeat expansion in the androgen receptor gene (La Spada, Wilson, Lubahn, Harding, & Fischbeck, 1991). Athletes especially those engaging in contact sports like Soccer/baseball which require a high degree of endurance have an increased susceptibility to ALS. Studies have found that both male and female athletes in aggressive contact sports have higher testosterone levels (Pillay, 2006). Some authors have hypothesized that individuals with high prenatal testosterone exposure self‐select into aggressive sports and occupations which require endurance (Wicks, 2012). Fondell, Fitzgerald, Falcone, O’Reilly, & Ascherio (2013) found an increased risk of ALS in men with early‐onset androgenic alopecia. Gargiulo‐Monachelli (2014) found that postmenopausal female ALS patients had significantly higher serum total testosterone and serum free testosterone concentrations than age‐matched postmenopausal controls. Studies have found a link between head trauma and ALS Chen, Richard, Sandler, Umbach, & Kamel (2007). This can be explained by the simple fact that many of these injuries occurred in those engaging in contact/collision sports and as mentioned in point 4, probably individuals with high testosterone self‐select into aggressive sports/jobs requiring physical exercise. Vivekananda et al. (2011) found that ALS patients had lower Ratio of the index and ring finger lengths (2D:4D ratio) in comparison with controls. 2D:4D ratio is dependent on prenatal testosterone levels. A study (Militello et al., 2002) of male and female patients of ALS found that serum free testosterone levels were significantly lower in both male and female ALS patients.

However, there also have been observations which did not directly implicate testosterone Bruson et al. (2012) analyzed Androgen Receptor CAG expansions in 336 patients with ALS and 100 controls and found no significant difference between the 2 groups. Jones, Riley, & Antel (1982) treated male ALS patients with high dose testosterone and found that exogenous high dose testosterone therapy caused a predictable decrease in basal LH and FSH levels and expected dampening of LH and FSH response to GnRH stimulation.

In our study, we measured concentrations of testosterone and its principal metabolite dihydrotestosterone in cerebrospinal fluid (CSF) of ALS patients and compared it with those of normal controls.

## 2 METHODS

CSF levels of free testosterone and dihydrotestosterone were measured in 13 ALS patients [ 7 males and 6 females] and in 22 controls [12 males and 10 females]. All CSF samples were collected in the morning between 9:00 a.m. and 11:00 a.m. as some studies (Goodman, Hotchkiss, Karsch, & Knobil, 1974) have shown that testosterone concentrations vary during the day. Due clearances from Institutional Research and Ethical Committees was obtained. Written informed consent was taken from patients and controls for CSF and clinical data collection.

Inclusion criteria used for patient selection:

a) Patients fulfilling the diagnosis of clinically definite ALS and clinically probable ALS as per the El Escorial Criteria (EEC) (Brooks, Miller, Swash, & Munsat, 2000).

Inclusion criteria used for control selection:

a) Controls were enrolled from surgery, gynecology and obstetrics and from orthopedics wards. CSF was obtained from male and female controls undergoing lumbar puncture for spinal anesthesia for surgery.

Exclusion criteria used for both patient and control selection:

(a) Subjects not giving consent for study. (b) Subjects suffering from cryptorchidism, testicular malignancy or any other testicular pathology. (c) Subjects taking anabolic hormones. (d) Subjects suffering from pituitary or adrenal disease. (e) Female subjects with history of PCOD or history of hirsuitism. (f) Subjects with family history of ALS or any form of motor neuron disease or frontotemporal dementia [FTD].

CSF concentrations of free testosterone and dihydrotestosterone were measured using solid phase enzyme‐linked immunosorbent assay (ELISA) based kits sourced from IBL International.

## 3 RESULTS

**Figure 1:**
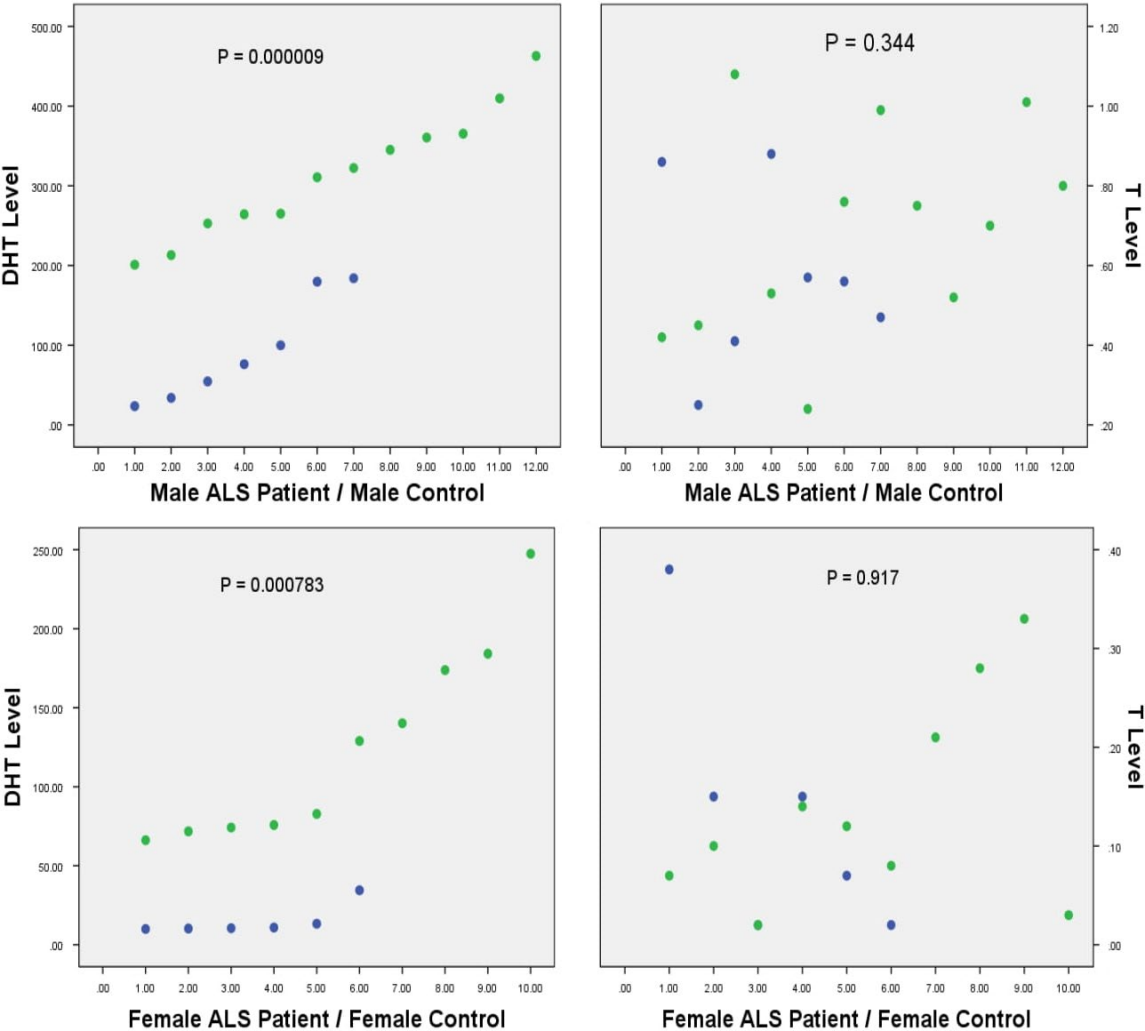
CSF Dihydrotestosterone and CSF Testosterone concentrations in male and female ALS patients and controls compared. DHT‐Dihydrotestosterone, T‐ Testosterone, ALS – Amyotrophic lateral sclerosis. Blue dots indicate male and female ALS patients, green dots indicate male and female controls. Plotted on Y axis are levels of Dihydrotestosterone and Testosterone, on X‐ axis are plotted the study groups‐ ALS patients and controls

Clinical and demographic data and CSF concentrations of free testosterone and dihydrotestosterone in ALS patients and controls are summarized in Tables 1, 2, 3, 4, 5 and Figure 1.

**Table 1:** Age distribution of ALS patients and controls

ALS patients		Controls	
	Mean Age and range(years)		Mean Age and range(years)
Males	51.57 (30–60)	Males	44.75 (19–62)
Females	59.33 (50–68)	Females	46.50 (25–71)

**Table 2:** Clinical details of Male ALS patients

Age (years)	Clinical Symptoms	Comorbidities, Drugs being taken	Symptom duration at presentation to our institute	CSF	MRI Brain and Cervical spine
58	right upper limb weakness f/b left UL weakness f/b right LL weakness f/b bulbar symptoms	Bronchial Asthma. Salbutamol inhaler, Riluzole	1 years 3 months	P−44 G−36 Cells‐ 2 L	Normal
30	right upper limb weakness f/b right LL weakness f/b left LL weakness, no bulbar symptoms. Tongue fasciculations present.	None Riluzole	2 years 5 months	P−40 G−56 Cells‐Acellular	Mild Fronto‐parietal atrophy
45	left UL weakness with atrophy f/b right UL weakness	COPD, alcoholism,old treated PTB 6 years back. Riluzole	1 year	P−32.6 G−57 Cells−0	Frontal atrophy
54	Right lower limb weakness f/b left LL weakness f/b right UL weakness for 1 ½ year with bulbar symptoms for 4 months.	HTN,DCM Beta blockers, ACE inhibitors,Diuretics, Riluzole, Edaravone	1 ½ year.	P−48 G−51 Cells−0	White matter periventricular hyperintensities.
60	florid fasciculations f/b right UL wasting f/b left UL wasting f/b left LL wasting.	Riluzole	8 months	P−32 G−44 Cells−3L	Frontal atrophy
59	bulbar onset weakness f/b UL weakness f/b LL weakness	HTN Diuertics, Riluzole	1 ½ year	P−26.2 G−51 Cells−0	normal
55	fasciculations over proximal arms, shoulders f/b dysarthria for 3 months, right UL weakness for 3 months, left UL weakness for 2 months.	none	6 months	P−28.9 G−62 Cells‐Acellular	normal

P‐Total CSF protein, G‐Glucose, L‐lymphocytes.

**Table 3:** Clinical details of Male Controls

Age (years)	Surgical Procedure Undergone	Comorbidities
50	Stapler hemorroidectomy for hemorrhoids	COPD, reformed Alcoholic.
61	Ureteric stricture reconstructive surgery	HTN x 5 years
60	Right tibia fracture	—
24	Right ACL tear arthroscopic surgery	—
26	Fistula‐in‐ano surgery	—
19	ACL tear arthroscopic surgery plus bucket handle tear of medial meniscus	—
22	Right ACL tear arthroscopic surgery	—
35	Fournier Gangrene	None
60	TURBT – Transurethral resection of bladder tumor	—
60	Cholecystectomy for Cholelithiasis	Hyperlipidemia with HTN
62	Ureteroscopy for urolithiasis	—
58	Exploratory laprotomy	HTN

**Table 4:** Clinical details of female ALS patients

Age (years)	Clinical Symptoms	Comorbidities,Drugs being taken	Symptom duration at presentation to our institute	CSF	MRI Brain and Cervical spine
50	Proximal weakness in right lower limb for 11 months f/b dysarthria and left lower limb weakness for 7 months.	Riluzole	11 months	P−46 G−68 Cells −0	T2/FLAIR HI post limb IC with decreased fractional isotropy on DTI in B/L CST.
68	Distal weakness in B/L upper limb for 1 ½ year f/b neck drop for 6 months f/b bulbar symptoms for 4 months.	Hypothyroidism for 30 years. Thyroxine, Riluzole	1 ½ year	P−32 G−57 Cells−2L	normal
65	Left UL weakness for 6 months f/b left LL weakness for 4 months	Riluzole	6 months	P−30 G−64 Cells −0	Mild frontal atrophy
60	Slurring of speech for 6 months with neurogenic dysphagia.	HTN. ACE inhibitors,Riluzole	6 months	P−42 G−62 Cells−4L	white matter arteriosclerotic Hyperintensities.
62	B/L proximal lower limb weakness with wasting for 1 year f/b distal lower limb weakness for 7 months f/b development of florid fasciculations and bulbar symptoms for 4 months.	B/L mal rotated kidney on abdominal imaging. Riluzole	8 months	P−34 G−51 Cells−0	Fronto‐parietal atrophy
51	Difficulty in speaking with nasal regurgitation of liquids for 5 months, no limb weakness.	DM. MetforminglimepirideRiluzole.	5 months	P−44 G−60 Acellular	Normal

P‐Total CSF protein, G‐Glucose, L‐lymphocytes

**Table 5:** Clinical details of female controls

Age (years)	Surgical Procedure	Comorbidities
56	Left Acetabular Fx Surgery	
40	Recto‐vaginal fistula repair	
71	Right total hip replacement.	HTN
45	Left ACL tear repair.	
35	Tubal Surgery	VSD
57	Exploratory Laprotomy	
35	Uterine surgery	Hypothyroidism
36	Uterine surgery	DM with Hypothyroidism
25	Arthroscopic right lateral parameniscal cyst excision	
65	Vaginal hysterectomy	

The data sets were found to have a normal distribution using Kolmogorov–Smirnov [K‐S] goodness‐of‐fit test. Thus, we applied students *t* test to evaluate whether there was any significant difference between the 2 groups.

Results are summarized as

CSF testosterone values in Male ALS patients and controls ‐No significant difference [*p* = .344].

CSF dihydrotestosterone values in Male ALS patients and controls—Significant difference was found with values significantly less in ALS patients.[*p* = .000009]. CSF testosterone values in female ALS patients and controls‐No significant difference [*p* = .917]. CSF dihydrotestosterone values in female ALS patients and controls—significant difference was found with values significantly less in ALS patients [*p* = .000783].

Thus, we see that CSF free testosterone levels did not show any significant differences in both male and female ALS patients as compared to those of male and female controls. However, CSF dihydrotestosterone levels were significantly decreased both in male and female ALS patients as compared with those of male and female controls.

## 4 DISCUSSION

In our study, we have demonstrated significantly decreased CSF dihydrotestosterone levels in both male and female ALS patients. More importantly, all ALS patients studied had decreased CSF dihydrotestosterone pointing to this being an important contributing factor to ALS pathogenesis.

There have been few studies done on CSF penetration and CSF metabolism of testosterone and dihydrotestosterone and how testosterone and its metabolites exert a negative influence on LH release. These studies have demonstrated the following observations.

Radioactively labeled dihydrotestosterone was given intravenously to six adult male rhesus monkeys (Macaca mulata), and it was found that that almost no radioactively labeled DHT could be found in CSF postinjection thus demonstrating that dihydrotestosterone has minimal penetration across the blood–brain barrier [BBB] (Marynick, Havens, Ebert, & Loriaux, 1976). A study (Abbott, Batty, Dubey, Herbert, & Shiers, 1985) on castrated male monkeys found that even in presence of very high, supraphysiological serum levels of DHT, CSF concentrations of DHT stayed very low. Also, there was no correlation between levels of unbound fraction of DHT in serum and DHT in CSF despite the fact that in CSF, the entire DHT fraction is unbound. The authors hypothesized that some special mechanism or carrier protein limits the influx of DHT in CSF from serum even in presence of very high supraphysiological DHT serum concentrations (Abbott et al., 1985). In another study (Schaison, Renoir, Lagoguey, & Mowszowicz, 1980), it was found that administration of DHT was unable to suppress LH in either normal men or agonadal patients. DHT was administered through the percutaneous route as a hydrosoluble gel for 3 months to all subjects and plasma DHT levels 8–10 times the normal plasma range were reached and maintained without any reduction in circulating LH levels. This study demonstrated that DHT is unable to enter the CSF compartment despite the presence of sustained high, supraphysiological serum levels. The above findings are in contrast to CSF penetration dynamics of testosterone. Studies (Backstrom, Carstensen, & Sodergard, 1976; Dubey, Herbert, Abbott, & Martensz, 1984) have shown that CSF levels of testosterone are equal to those of unbound serum testosterone levels with the entire CSF testosterone being unbound and that there is a predictable relationship between serum total testosterone levels, serum unbound testosterone levels and testosterone levels in the CSF. Also, it is known that testosterone crosses the BBB (blood brain barrier) only in the unbound state. These studies also found that serum testosterone concentrations show a pronounced diurnal variation and that in castrated animals, concentrations similar to the high early morning serum concentrations in the precastrated state could only be achieved with much higher doses of testosterone. The study (Dubey et al., 1984) also found that a sharp increase in serum total testosterone, unbound serum testosterone and CSF testosterone coincided with an abrupt fall in serum LH (Luteinising hormone) levels. Abbott et al. (1985) demonstrated in their study that DHT can suppress serum LH levels and also concurred with a previous study by Sholl, Goy, & Uno (1982) that a majority of DHT within the brain comes from the precursor testosterone. A study (Martini, Celotti, & Melcangi, 1996) using fetal and neonatal rat brain cells found that formation of DHT takes place preferentially in the neurons in the nervous system although type‐2 astrocytes and oligodendrocytes also possess some 5 alpha‐reductase activity. Other studies (Celotti, Melcangi, Negri‐Cesi, & Poletti, 1991) have also echoed similar findings. Pardridge, Moeller, Mietus, & Oldendorf (1980) in their study found that DHT is sequestered to a greater degree in mammalian brains than is testosterone. Moreover, it is a known fact that DHT is a much more potent hormone than testosterone because its binding affinity to the androgen receptor is two times that of testosterone and it has a dissociation rate about a fifth of that of testosterone (Marchetti & Barth, 2013). Sholl, Robinson, & Goy (1975) from their study concluded that DHT is the primary androgen for activation of neural mediated effects, at least in the guinea pig. In a study done by Zoppi et al. (1988) on rats, it was found that when testosterone was given along with an inhibitor of 5 alpha‐reductase thereby leading to lesser conversion of testosterone to DHT, LH levels were reduced to a lesser extent than they were when testosterone was given alone. Also, it has been found that patients with 5 alpha‐reductase type 2 deficiency have high circulating LH levels despite having normal or elevated serum levels of testosterone. However, these patients have reduced serum DHT levels (Martini, Celotti, & Serio, 1979). Various other analyses (Zanisi, Motta, & Martini, 1973) have also shown that DHT and its metabolite, 3‐alpha *diol* are much more effective that testosterone in suppressing LH secretion. A study evaluating effects of testosterone,DHT and its metabolites in cultured pituitary cells (Denef, 1983) provided further evidence for a specific physiological role of 5‐DHT and 3 ‐alpha diol. This study concluded that at least at the gonadotroph cell level, DHT and possibly 3‐alpha diol are the active androgens which depress LH release. Another study (Kennedy, Rawlings, & Cook, 1985) done on bull calves also found that LH serum concentrations were suppressed by administration of sialistic implants releasing DHT and also by 3‐alpha diol implants. Studies (Martini, 1982) have found that the anterior pituitary very efficiently metabolises testosterone to DHT and 3α‐diol. Yields of DHT generated are second only to yields seen in the prostate gland. Also, in the anterior pituitary, around 25% of testosterone is converted into 3‐alpha diol while in the prostrate, only 7.6% of testosterone is converted into 3‐alpha diol. It has also been observed that the anterior pituitary of adult mammals does not have significant aromatizing ability while the hypothalamus has potent aromatizing abilities. Studies (Chimento, Sirianni, Casaburi, & Pezzi, 2014) suggest that estradiol derived from intracerebral testosterone is the main hormone that provides negative feedback at the hypothalamic level while at the level of the anterior pituitary, both DHT and estradiol, both derived from intracerebral testosterone, are required for negative LH feedback. Numerous animal studies (Abdelgadir et al., 1994; Scott, Kuehl, Ferreira, & Jackson, 1997) also support that estradiol derived from intracerebral testosterone is the main hormone that provides negative feedback at the hypothalamic level while at the anterior pituitary level, DHT also plays a very important role in suppressing LH. Interventions like reducing DHT levels with either a reductase inhibitor or by using antibodies to estradiol or through administration of aromatase inhibitors—all these maneuvers compromise the ability of T to suppress LH.

Interestingly, the anterior pituitary and the hypothalamic/preoptic area possess 5 α reductase type 2 isoenzyme whereas the glial cells and the neurons predominantly express the 5 α reductase type 1 isoenzyme. It was also found that in the rat pituitary, 5 α reductase type 2 isoenzyme is located mainly in the gonadotropes. Various authors have concluded that type 1 5 α reductase is constitutively expressed in the mammalian brain and appears to have a neuroprotective role and mutations in type 1 5 α reductase lead to death of the organism in gestation itself (Mahendroo, Cala, Landrum, & Russell, 1997; Poletti et al., 1998). As mentioned above, patients with 5 alpha‐reductase type 2 deficiency have high circulating LH levels, normal or elevated serum levels of testosterone and reduced serum DHT levels. However, these patients do not have an increased risk of ALS. Total testosterone and free non‐SHBG bound testosterone have been found to have a diurnal circadian variation with highest levels being seen in the morning and lowest in the evening (Bremner, Vitiello, & Prinz, 1983; Plymate, Tenover, & Bremner, 1989; Tenover, Matsumoto, Clifton, & Bremner, 1988). A study (Bremner et al., 1983) documented that elderly males have reduced testosterone levels as compared to younger males but more importantly, there was a greater, more pronounced difference between the circadian excursion of total serum testosterone levels between the two groups. Plymate et al. (1989) found that elderly males have a reduced significant circadian rhythm in free non‐SHBG bound testosterone as compared to younger males [60% versus 100%]. This study further found that even in 60% of older males showing circadian variation in free non‐SHBG bound testosterone, the circadian excursion was only 26% of what was seen in the younger males. Tenover et al. (1988) found that the circadian pattern of pulsatile LH secretion was blunted in healthy, elderly males as compared to young males. Studies (Gapstur et al., 2002; Leifke, Gorenoi, Wichers, Von Zur, & Brabant, 2000) have consistently shown that both total testosterone and free testosterone concentrations decrease with ageing while SHBG levels increase with ageing. Studies (Laaksonen et al., 2004) have shown that obesity, insulin resistance, metabolic syndrome and dyslipidemia have a strong association with low serum levels of total testosterone, free testosterone and sex hormone binding globulin (SHBG). In addition, it was found (Laaksonen et al., 2004) that the association between low levels of free testosterone and diabetes was abolished and that between low levels of total testosterone and diabetes was attenuated after adjusting for BMI (Body Mass Index) while in case of SHBG, the association was unaffected. This study suggested that SHBG levels plays a more important role in the development of insulin resistance/metabolic syndrome than total or free testosterone does. Many studies (Scarmeas, Shih, Stern, Ottman, & Rowland, 2002) have suggested that ALS patients have had a lower premorbid body mass index as compared to controls and have been leaner, fitter in their life as compared to controls. In other words, being obese or having a higher BMI is protective against development of ALS in later life. Studies (Reich‐Slotky et al., 2013) have found that having a higher premorbid BMI predicts a better result on the ALS Functional Rating Scale (ALSFRS‐R) meaning that overweight/obese persons even after developing symptoms and signs of ALS fare better than their leaner counterparts ALS patients having higher subcutaneous fat had increased duration of survival after developing the disease. There is emerging evidence that high carbohydrate/high fat hypercaloric diets may improve survival in ALS patients. In a clinical trial (Wills, Hubbard, & Macklin, 2014) on 24 ALS patients utilizing hypercaloric high carbohydrate diet, it was found that the high carbohydrate diet group had a longer survival than the placebo group (0% death versus 43% death, respectively, after five months of follow‐up). Studies in animal models of ALS have demonstrated improved survival with high fat high calorie diets. Breedlove & Arthur (1983) in their study on motor neurons found that following systemic administration of radioactive androgens, DHT accumulated to a greater degree in the spinal motor neuron nuclei than testosterone and unlike testosterone, DHT concentrations in spinal motor neurons did not show any sex difference. This shows that DHT is an essential, integral component of sex steroid machinery in the motor neurons regardless of sex of the organism.It was earlier widely believed that the hypothalamic–pituitary–gonadal axis in humans remains quiescent after birth for around 10 years until the onset of pubertal activation and that LH and FSH night‐day rhythms begin just before the onset of puberty, triggering its onset. This erroneous assumption probably stemmed from the higher detection limits of the hormonal assays used in the previous studies (Mitamura et al., 1999). However, studies using ultra‐sensitive assay methods have shown that circulating gonadotrophin concentrations and diurnal Rhythms of Luteinizing Hormone, Follicle‐Stimulating Hormone, Testosterone, and Estradiol Secretion exist and are well established in prepubertal children as young as 4.6 years One is justified in making an assumption based on these findings that probably biorhythms of these hormones exist in younger children too. Studies on animal models of ALS (McLeod et al., 2019; Yoo & Ko, 2012) have also shown androgens deficiency/receptor antagonism may contribute to ALS.

Based on data from the above studies, we postulate the following hypothesis‐DHT or one of its metabolites is probably integral to survival of motor neurons and in ALS, it is lack of DHT in the motor neurons which leads to their death. CSF levels of DHT are probably the final arbiter of LH release at the level of anterior pituitary. Entire DHT fraction in the central neural axis is derived from the testosterone fraction which penetrates the BBB since DHT itself cannot penetrate BBB. Dubey et al. (1984) had found that a sharp increase in serum total testosterone, unbound serum testosterone and CSF testosterone had coincided with an abrupt fall in serum LH (Luteinising hormone) levels. We postulate that when high testosterone concentrations are reached and they breach a certain “critical” threshold, the binding capacity of SHBG (Sex hormone binding globulin) is exceeded and only then does the levels of free, unbound serum testosterone rise sufficiently to cause the rise in CSF testosterone [ in CSF, all of testosterone is free and unbound]. This CSF testosterone is converted to dihydrotestosterone and the resultant increase in dihydrotestosterone suppresses LH levels by exerting a negative feedback on LH release. We postulate that in patients who are predisposed to develop ALS, there is a sort of “testosterone resistance” at the level of BBB. This “testosterone resistance” exists right from birth. This “testosterone resistance” is likely to be the result of a faulty, mutated transport protein involved in testosterone transfer across the BBB. In these patients, lesser amount of testosterone is able to breach the BBB and enter the central neural axis. As a result, lesser amount of testosterone is available to 5 α reductase type 2 isoenzyme in the anterior pituitary to be converted to DHT and lesser amount of DHT is generated. As a result, there is inadequate negative feedback suppression of LH at the level of anterior pituitary by DHT or its metabolites like 3‐alpha diol. As a result of higher LH levels, testosterone levels rise in the peripheral testosterone fraction [the fraction outside the BBB] and this explains the various physical attributes of ALS patients like the lower Ratio of the index and ring finger lengths (2D:4D ratio), increased incidence of early‐onset androgenic alopecia, the increased athleticism in the premorbid years, the lower BMI in the premorbid years (the “always been lean”) phenomenon etc. It is also possible that the higher peripheral concentrations of testosterone in ALS patients are a sort of compensatory mechanism of the human body to protect the motor neurons against DHT deprivation. Higher concentrations of testosterone in the peripheral pool would translate in higher amounts of testosterone being available at the “testosterone resistant” BBB as compared to normal individuals with normal BBB and even with less than normal levels entering the central neural axis as compared to normal individuals with normal BBB [ Although this would exactly depend on the morning peak testosterone levels], these high peripheral free testosterone levels would in some degree compensate for the “testosterone resistance” at the level of BBB and maintain the DHT levels in the central neural axis and motor neurons essential to their survival. As long as these compensatory mechanisms worked, those with the BBB “testosterone resistance” would not develop signs of ALS. However, when these compensatory mechanisms would start failing and the high peripheral pool testosterone concentrations would start decreasing, the amounts of testosterone traversing the “testosterone resistant” BBB would decrease, the free testosterone entering the central neural axis would decrease, and as a result, the concentration of DHT in central neural axis and in the motor neurons would fall leading to their death and ALS would then set in. This fail in the compensatory mechanisms can be because of many reasons. For example, with advancing age, the circadian excursion in free non‐SHBG bound testosterone would decrease leading to lesser free testosterone being available at the BBB for intracerebral transport and this in a person having BBB “testosterone resistance” would translate in ALS initiation as in their case, the amount of testosterone crossing the BBB in proportion to their highest morning testosterone levels would be already low as compared to normal persons and any decrease in free testosterone levels in the central neural axis would probably also make the DHT levels in the central neural axis go below the critical threshold limit.Probably this is the reason ALS incidence increases with increasing age. We also hypothesise that persons developing ALS at younger age have a greater degree of “testosterone resistance” at the BBB and in this subset, the compensatory mechanisms fail sooner as compared to those developing ALS at a later age who would probably have a lesser degree of “testosterone resistance” at the BBB. Some evidence for the above proposed theory already exists in observations of Gargiulo‐Monachelli (2014) who found higher total and free testosterone levels in ALS patients who had a more rapid decline in their clinical status. Probably these patients had higher “testosterone resistance” at the BBB, lower DHT levels in anterior pituitary, higher LH release and resultant higher total and free testosterone levels. Our hypothesis would also justify some studies which have proposed that high carbohydrate/high fat hypercaloric diets improve survival in ALS patients. Probably these dietary interventions lead to lowering of serum SHBG levels. This in turn increases serum free testosterone levels and more free testosterone was available at the “testosterone resistant” BBB leading to higher DHT levels in the central neural axis and improved motor neuron survival. The same explanation suffices for the fact that overweight/obese persons even after developing symptoms and signs of ALS fare better than their leaner counterparts owing to their lower SHBG levels and higher free testosterone levels. We have proposed that reduced levels of DHT or its metabolites secondary to a dysfunctional BBB which has “testosterone resistance” is the primary cause of ALS. Reduced levels of DHT can be either because of substrate deficiency, enzyme deficiency or dysfunction and receptor dysfunction. However, we have kept substrate deficiency, that is decreased amount of free testosterone in the central neural axial compartment as the first probability owing to these reasons. The substrate deficiency in ALS is age dependent and is dependent on the degree of “testosterone resistance” of the faulty BBB. Till the high peripheral testosterone levels are maintained, substrate deficiency does not set in but as soon as the peripheral testosterone levels fall, there ensues deficiency of free testosterone in the central neural axis for conversion to DHT. Both 5 α reductase isoenzymes are apparently normal in ALS patients. In addition to reasons already mentioned, it is unlikely that enzyme dysfunction is at fault because of usual late age of onset of ALS and that ALS symptoms stay confined to the neural axis while the enzymes are widespread across many systems of the human body**.** For the same reasons, receptor dysfunction is unlikely to be the culprit. In addition, a study (Bruson et al., 2012) found normal androgen receptor function in ALS. More studies evaluating these parameters and also concurrent serum testosterone, serum dihydrotestosterone and LH levels would shed more light on ALS pathogenesis.

Conclusions—Our study implicates dihydrotestosterone deficiency in the neural axis as an important component of ALS pathogenesis. More studies evaluating these parameters and also concurrent evaluation of serum testosterone, serum dihydrotestosterone and LH levels would shed more light on ALS pathogenesis. Long‐term serial studies on asymptomatic familial ALS gene carriers would be invaluable in delineating the pathogenesis of this dreaded disease.

## CONFLICTS OF INTEREST

None.

## AUTHORS CONTRIBUTION

Dr Nishit Sawal: Conceived the entire idea , recruited all patients , wrote the manuscript and uploaded it. Dr Jasbinder Kaur: Did the CSF ELISA testosterone and dihydrotestosterone evaluation of all patients and controls and co‐authored the manuscript. Kamaljeet Kaur: Did the CSF ELISA testosterone and dihydrotestosterone evaluation of all patients and controls and co‐authored the methods portion of the manuscript. Dr Satinder Gombar: Recruited Controls for the study. There is no conflict of interest for any of the authors. The authors take full responsibility for the data, the analyses and interpretation, and the conduct of the research; full access to all of the data; and the right to publish any and all data.

## Supporting information

Appendix S1

## Data Availability

The data that support the findings of this study are available on request from the corresponding author. The data are not publicly available due to privacy or ethical restrictions.
