# Supplementary material for: Dihydrotestosterone in Amyotrophic lateral sclerosis—The missing link?
Source: Brain Behav. 2020 Oct 13;10(11):e01645. doi: 10.1002/brb3.1645 (PMC7667368; doi:10.1002/brb3.1645)
Supplement: Supplementary file 1 — Appendix S1 [file BRB3-10-e01645-s001.docx]

**Supplemental Table – Clinical details and CSF testosterone/Dihydrotestosterone values of Familial ALS patients:**

| **Age**  **(years), Gender** | **Clinical Symptoms** | **Co-morbidities, Drugs being taken** | **Symptom duration at presentation to our institute** | **MRI Brain and Cervical spine.**  **Genetic evaluation** | **CSF Testosterone (pg/ml)** | **CSF Dihydrotestosterone (pg/ml)** |
| --- | --- | --- | --- | --- | --- | --- |
| **35, Female^1^** | **Cramps left lower limb f/b distal , pure motor weakness left lower limb f/b proximal left lower limb weakness with fasciculations** | **None,**  **Riluzole** | **8 months** | **Normal**  **SOD1 gene mutation;**  **c.131A>G (p.His44Arg).** | **0.14** | **78.8** |
| **24, Male^2^** | **right lower limb weakness f/b left Lower limb weakness f/b right upper limb weakness with bulbar symptoms .** | **None**  **Riluzole** | **2 years** | **Normal.**  **WES report awaited.** | **0.6** | **33.9** |

1. Patients father and older brother had similar illness.
2. Patients mother developed pure motor , distal weakness of right lower limb with fasciculations at age of 35 which progressed over 3 years to involve all 4 extremities with bulbar involvement. Patient’s mother died of aspiration pneumonia.

CSF-cerebrospinal fluid, ALS-amyotrophic lateral sclerosis, SOD1-superoxide dismutase 1 enzyme, WES- whole exome sequencing.
